# Supplementary material for: Comprehensive Annotation and Functional Exploration of MicroRNAs in Lettuce
Source: Front Plant Sci. 2021 Dec 24;12:781836. doi: 10.3389/fpls.2021.781836 (PMC8739914; doi:10.3389/fpls.2021.781836)
Supplement: Supplementary file 3 [file Data_Sheet_9.ZIP › GSM2230753.plot/Lsa-miR396e_Lsat_1_v5_gn_7_89080.1_1990_TPlot.pdf]

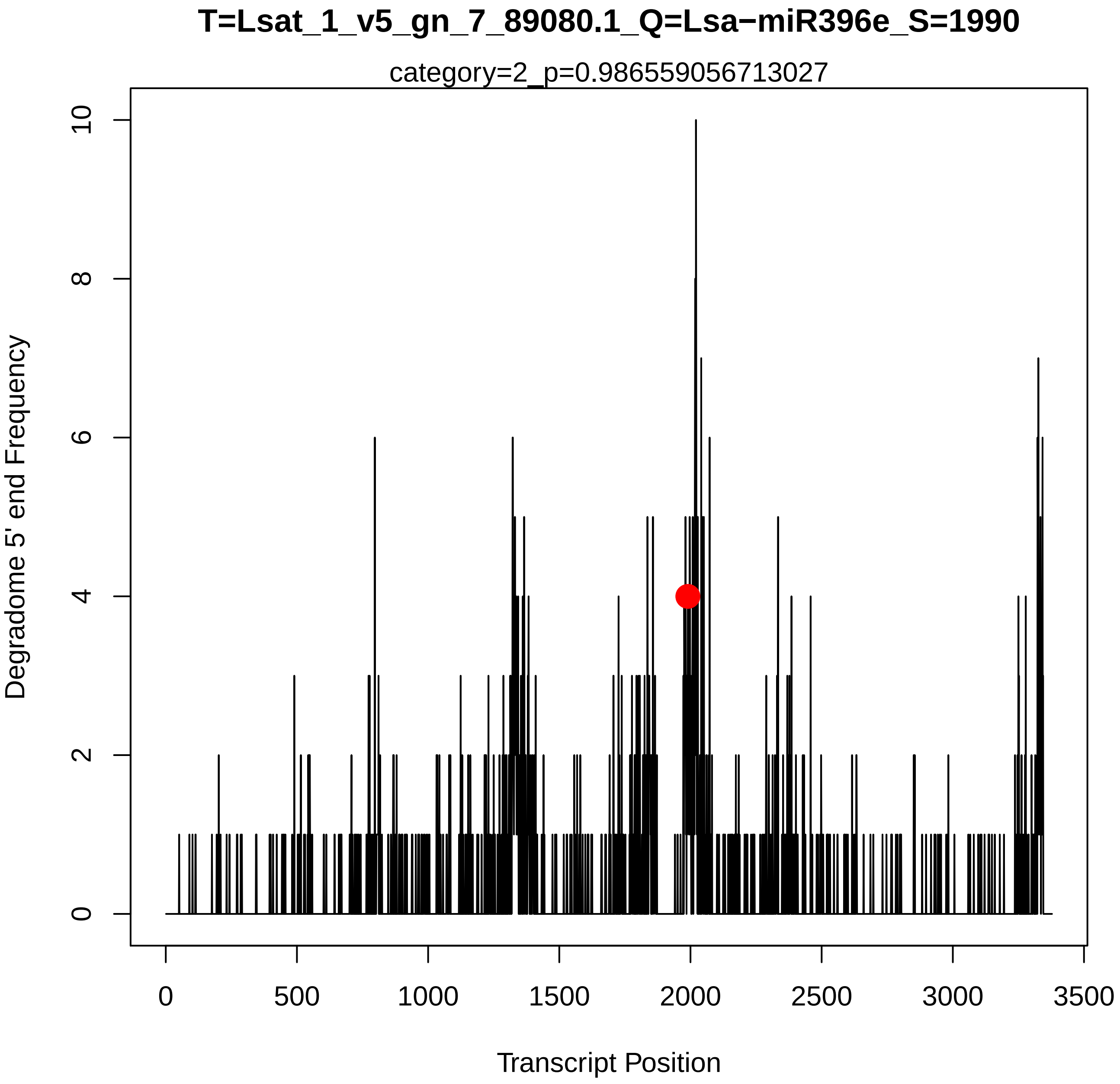
T=Lsat_1_v5_gn_7_89080.1_Q=Lsa−miR396e_S=1990
category=2_p=0.986559056713027
Degradome 5' end Frequency
0
2
4
6
8
10
0
500
1000
1500
2000
2500
3000
3500
Transcript Position
